# Supplementary material for: A blood-based signature of cerebrospinal fluid Aβ1–42 status
Source: Sci Rep. 2019 Mar 11;9:4163. doi: 10.1038/s41598-018-37149-7 (PMC6409361; doi:10.1038/s41598-018-37149-7)
Supplement: Supplementary file 1 — Supplementary Information [file 41598_2018_37149_MOESM1_ESM.pdf]

A blood-based signature of cerebrospinal fluid $A\beta_{1-42}$ status

Benjamin Goudey[1,2,3,*], Bowen J Fung[1,4,*], Christine Schieber[1], for the Alzheimer's Disease Metabolomics Consortium[5], for the Alzheimer's Disease Neuroimaging Initiative[6], and Noel G Faux[1,7,+]

[1]IBM Australia - Research, Carlton, Victoria, Australia
[2]Centre for Epidemiology and Biostatistics, The University of Melbourne, Parkville, Australia
[3]Department of Computing and Information System, The University of Melbourne, Parkville, Australia
[4]School of Psychological Sciences, University of Melbourne, Victoria, Australia
[5]A complete listing of ADNI investigators can be found at: http://adni.loni.usc.edu/wp-content/uploads/how_to_apply/ADNI_Acknowledgement_List.pdf.
[6]A complete listing of ADMC investigators can be found at: https://sites.duke.edu/adnimetab/who-we-are/
[7]The Florey Institute for Neuroscience and Mental Health, The University of Melbourne, Parkville, Australia
[+]Corresponding author: noel.faux@au1.ibm.com
[*]These authors contributed equally to this work

1 Supplementary methods

1.1 Download date

The data used in this study were obtained from the Alzheimer's Disease Neuroimaging Initiative (ADNI) database (http://adni.loni.usc.edu/), downloaded 10th October 2017 as the R package, ADNIMERGE.

1.2 Plasma protein level measurements

Plasma samples were analyzed by Rules-Based Medicine (RBM) (Austin, TX) using the Human DiscoveryMAP multiplex immunoassay[1]. This immunoassay panel of 190 analytes (Table 'rbmqc' in ADNIMERGE) included proteins previously reported to be involved in cell-signaling and/or associated with a variety of disease processes, including AD, metabolic disorders, inflammation, cancer, and cardiovascular disease. Of the 190 analytes, 146 met previously described quality control procedures (details at http://adni.loni.usc.edu/wp-content/uploads/2010/11/BC_Plasma_Proteomics_Data_Primer.pdf). We also examined total plasma homocysteine (Table 'hcres' in ADNIMERGE) and $A\beta_{1-40}$ and $A\beta_{1-42}$ levels in plasma (Table 'upennplasma' in ADNIMERGE) for each patient, given these have previously been associated with increased Alzheimer's disease risk.

1.3 Plasma metabolite level measurements

Metabolites from plasma samples were measured using the AbsoluteIDQ-p180 kit using an ultra-performance liquid chromatography system. This targeted metabolomics approach led to measures of 186 endogenous metabolites (Tables 'admcdukep180fia' and 'admcdukep180uplc' from ADNIMERGE) quantitatively (amino acids and biogenic amines) and semiquantitatively (acylcarnitines, sphingomyelins, PCs, and lyso-glycero-phosphatidylcholines across multiple classes). A multi-stage process was developed by the Alzheimer's Disease Metabolomics Consortium (ADMC) to remove missing data, duplicated and generally poorly measure metabolites, resulting in 138 metabolites that pass this stringent QC[2]. In this work, we made use of the QC'd tables provided by ADMC in the supplementary methods of their related publication[2].

1.4 CSF Aβ_{1-42} measurements

CSF Aβ_{1-42} was measured using a Luminex xMAP platform and Innogenetics INNO-BIA AlzBio3 immunoassay (Table 'upennbiomk2' in ADNIMERGE), as described previously[3].

1.5 APOEε4 measurement

APOEε4 carrier status was genotyped using DNA extracted by Cogenics from a 3 mL aliquot of EDTA blood[4].

1.6 Data preparation

Any variables that had more than 15% missingness were removed from the analysis. The pattern of missingness for remaining variables was checked across the three diagnosis groups and no clear trend could be observed. No individual had more than 5% of variables marked as missing. All remaining missing data points were imputed using an unsupervised RF approach[5].

1.7 Univariate analysis

For the baseline analysis (Table 1 in the main paper), we evaluated the univariate association of demographic variables, *APOE*ε4 carrier status and CSF Aβ_{1-42} levels with diagnosis as well as normal, abnormal CSF Aβ_{1-42} categories. For continuous variables, analysis of covariance (ANCOVA, type II) analysis with post hoc linear hypothesis testing (Tukey comparison between groups) was performed. Normality of variables was checked by visual inspection of the histograms. Box-Cox analysis of the variables was also performed to find the most appropriate power transformation. For CSF Aβ_{1-42}, a square root transformation was required. For the analyses of the CSF measurements, *APOE*ε4 carrier status was included as a covariate. For categorical variables, chi-squared tests were performed.

1.8 Random Forest

Random Forests (RF) were selected as the modeling approach to predict CSF Aβ_{1-42} levels, for both the binary and regression tasks. RFs are a widely used ensemble method that builds many simple classifiers (decision trees) in the training phase, each constructed over different sample "bags", a set of samples randomly selected with replacement, and different subsets of features. After training, predictions for unseen samples are made by averaging over all individual trees, taking either the mean or the mode for regression or classification tasks respectively. RFs have a number of advantages for the small sample size and disparate types of features observed in the ADNI dataset. RFs are invariant to the scale of the observed features and make few assumptions about the distributions of observed data allowing them to be applied to multiple data modalities easily.

1.9 Metrics used to determine regression cutoff

Six criteria from the the R package *OptimalCutoffs*[6] were used to determine the optimal threshold for the continuous regression predictions. These criteria were:

- MaxProdSpSe - maximizes the product of sensitivity and specificity.
- NPVEqualPPV - finds the threshold whereby the negative and positive predictive value are closest.
- ValueSe - optimizes for a Sensitivity of 0.85.
- ValueSp - optimizes for a Specificity of 0.85.
- MinPvalue - minimizes the p-value from a Chi-squared test between the actual and predicted markers.
- PrevalenceMatching - matches prevalence estimates from actual and predicted labels.

These methods were selected as they represent the three classes of methodologies in the *OptimalCutoffs* package and were intuitive choices. Further choices were restricted due to concerns of runtime and overfitting.

1.10 All R packages used

All analysis was performed with R (3.3.2)[7], and used the following packages:

- RandomForestSRC[8]
- ggplot2[9]
- pROC[10]
- OptimalCutpoints[6]
- Survminer[11]
- Mixtools[12]
- Stargazer[13]
- ggExtra[14]
- stringr[15]
- dplyr[16]
- tidyr[17]
- pbapply[18]
- pander[19]
- data.table[20]
- gridExtra[21]
- caret[22]

1.11 Cohort Breakdown

Diagnosis	CN	MCI	AD
N	58	396	112
Mean age (sd)	75.11 (5.77)	74.74 (7.40)	74.80 (8.08)
N Females (%)	28 (48.28)	140 (35.35)	47 (41.96)
Mean years education (sd)	15.65 (3.04)	15.64 (3.04)	15.09 (3.20)
N *APOEε4* carriers (%)	5 (8.62)	211 (53.28)	76 (67.86)
N with CSF	58	198	102
Mean CSF Aβ_{1-42} (sd)	250.84 (21.08)	163.44 (52.76)	142.69 (39.15)
Mean CSF tTAU (sd)	63.62 (21.76)	102.33 (59.63)	120.94 (56.51)
Mean CSF pTAU (sd)	21.07 (8.43)	36.11 (19.27)	42.07 (19.87)

Table 1. Demographic characteristics of the ADNI data used, without stratification by whether CSF Aβ_{1-42} measures were available.

CSF Aβ_{1-42} category	Normal	Abnormal
N	119	239
Mean age (sd)	75.27 (7.22)	74.32 (7.40)
N Females (%)	44 (36.97)	92 (38.49)
Mean years education (sd)	15.75 (2.96)	15.52 (3.10)
N *APOEε4* carriers (%)	17 (14.29)	165 (69.04)

Table 2. Demographic characteristics of the training sub-cohort, stratified into CSF Aβ_{1-42} Normal/Abnormal using a threshold of $\leq$ 192 pg/mL for abnormal.

Diagnosis	CN		MCI		AD	
CSF Aβ_{1-42} category	Normal	Abnormal	Normal	Abnormal	Normal	Abnormal
N	58	0	53	145	8	94
Mean age (sd)	75.11 (5.77)	-	74.55 (8.31)	74.28 (7.18)	81.04 (7.31)	74.38 (7.77)
N Female (%)	28 (48.28)	-	12 (22.64)	53 (36.55)	4 (50.00)	39 (41.94)
Mean Years education (sd)	15.67 (2.78)	-	15.98 (3.06)	15.75 (2.97)	14.75 (3.69)	15.16 (3.27)
N *APOEε4* carriers (%)	5 (8.62)	-	12 (22.64)	94 (64.83)	0 (0.00)	71 (75.53)

Table 3. Demographic characteristics of the training sub-cohort, stratified by both baseline diagnosis and CSF Aβ_{1-42} Normal/Abnormal using a threshold of $\leq$ 192 pg/mL for abnormal.

2 Supplementary Results

2.1 Univariate analysis confirms no significant differences across diagnostic groupings

The cohort used in this study consisted of 58 cognitively normal (CN), 396 mild cognitively impaired individuals (MCI), and 112 subjects with Alzheimer's Disease (AD), with a mean follow up time of 4.1 years (2.9 SD). Table 1 in the main text presents the overall characteristics of the cohort, stratified by diagnosis as well as by subjects which have had CSF $A\beta_{1-42}$ measurements (training set) and those who did not (validation set). Univariate analysis across the diagnosis groupings (Supplementary Methods) confirms their similarity. There was no difference in age between the diagnostic groups ($p = 0.978$), nor was there a gender bias ($p = 0.104$). In line with previous studies, AD and MCI subjects had a greater number of *APOEε4* carriers, than CN ($p = 7.52 \times 10^{-13}, p = 4.97 \times 10^{-10}$, respectively). Further, there was no difference in the years of education between the diagnostic groups ($p = 0.219$). The AD subjects had lower CSF $A\beta_{1-42}$ compared to CN and MCI ($p < 2.221^{-16}, p < 2.221^{-16}$, respectively) subjects. These similarities were also observed within both the training and validation sets. However, across these two sets of individuals, there was a strong difference in the proportions of the diagnostic class, with the validation set containing 198 MCI but no CN and only 10 AD.

2.2 Performance of different feature subsets using only CN and MCI individuals

	Regression					Binary			
Feature set	AUC	R^2	Acc	Sens	Spec	AUC	Acc	Sens	Spec
BPM	**0.805 (0.07)**	0.241 (0.11)	**0.738 (0.07)**	**0.739 (0.13)**	0.732 (0.14)	**0.800 (0.07)**	**0.719 (0.07)**	**0.853 (0.09)**	0.543 (0.15)
BM	0.769 (0.09)	0.174 (0.13)	0.709 (0.07)	0.691 (0.14)	0.736 (0.14)	0.766 (0.08)	0.691 (0.08)	**0.841 (0.09)**	0.499 (0.15)
BP	**0.813 (0.08)**	**0.258 (0.12)**	**0.728 (0.08)**	**0.719 (0.13)**	0.735 (0.16)	**0.808 (0.07)**	**0.726 (0.08)**	**0.856 (0.09)**	0.552 (0.17)
B	0.759 (0.09)	0.229 (0.15)	**0.738 (0.08)**	0.690 (0.13)	**0.796 (0.15)**	0.765 (0.09)	**0.714 (0.08)**	0.698 (0.13)	**0.736 (0.16)**
BP_{fs}	0.787 (0.09)	0.224 (0.16)	**0.724 (0.08)**	**0.735 (0.13)**	0.704 (0.15)				

Table 4. Mean and standard deviation (in parentheses) of performance metrics (area under the receiver operator curve, AUC; accuracy, Acc; sensitivity, Sens; specificity, Spec and R^2 for the regression models) using individuals that were CN or MCI at baseline, for the different Random Forest models using different feature sets across all cross-validation folds. Left and right halves are for the Regression and Binary tasks respectively. Bold faced text on AUCs indicates the best performing model or those that are statistically equivalent (via a Wilcox rank signed test, with a Bonferroni-corrected significance threshold of 0.05/5=0.01). Features sets describe combinations of (B) baseline model (age and *APOE*ε4 carrier status), (P) Proteomics, (M) Metabolomics.

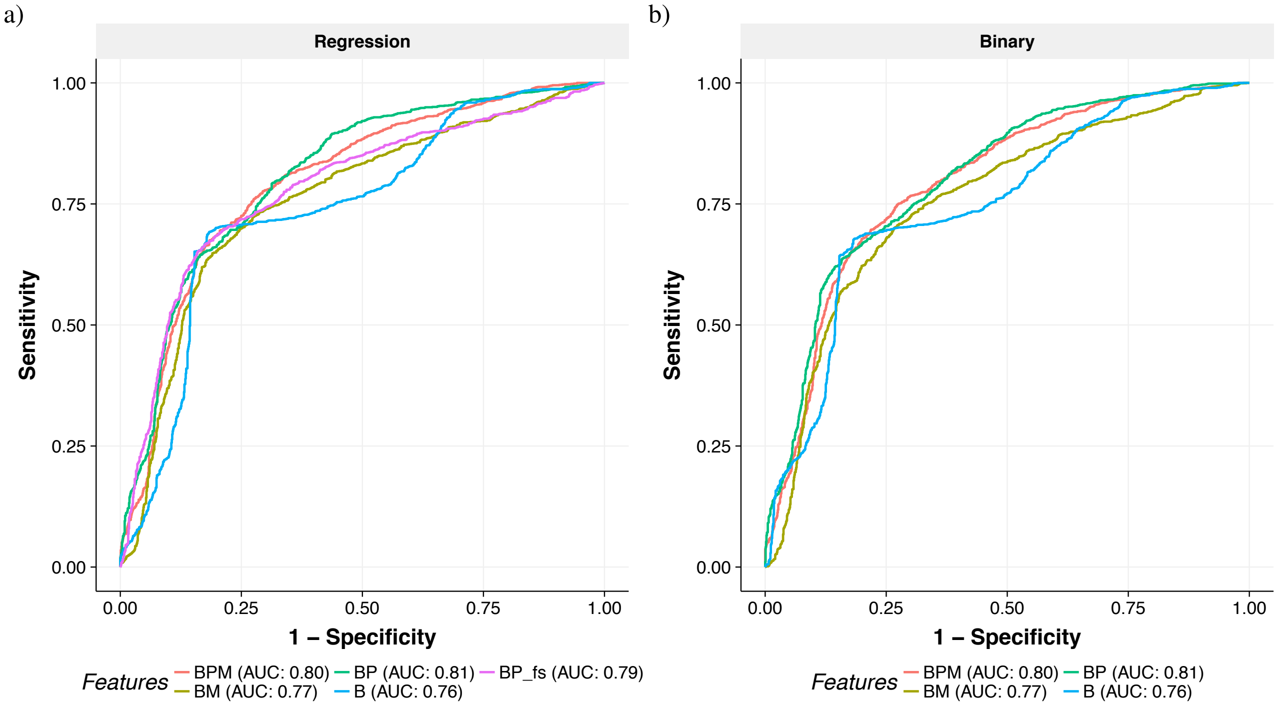

Figure 1. ROC curves derived on only CN and MCI individuals in the training cohort, comparing different sets of features to determine predictive value. a) regression, and b) binary. Different colours of lines correspond to different feature sets with corresponding AUCs indicating in the legend under each plot. Feature sets describe combinations of (B) baseline model (age and *APOE*ε4 carrier status), (P) Proteomics, (M) Metabolomics.

2.3 ROC curves for different feature subsets using only complete cases

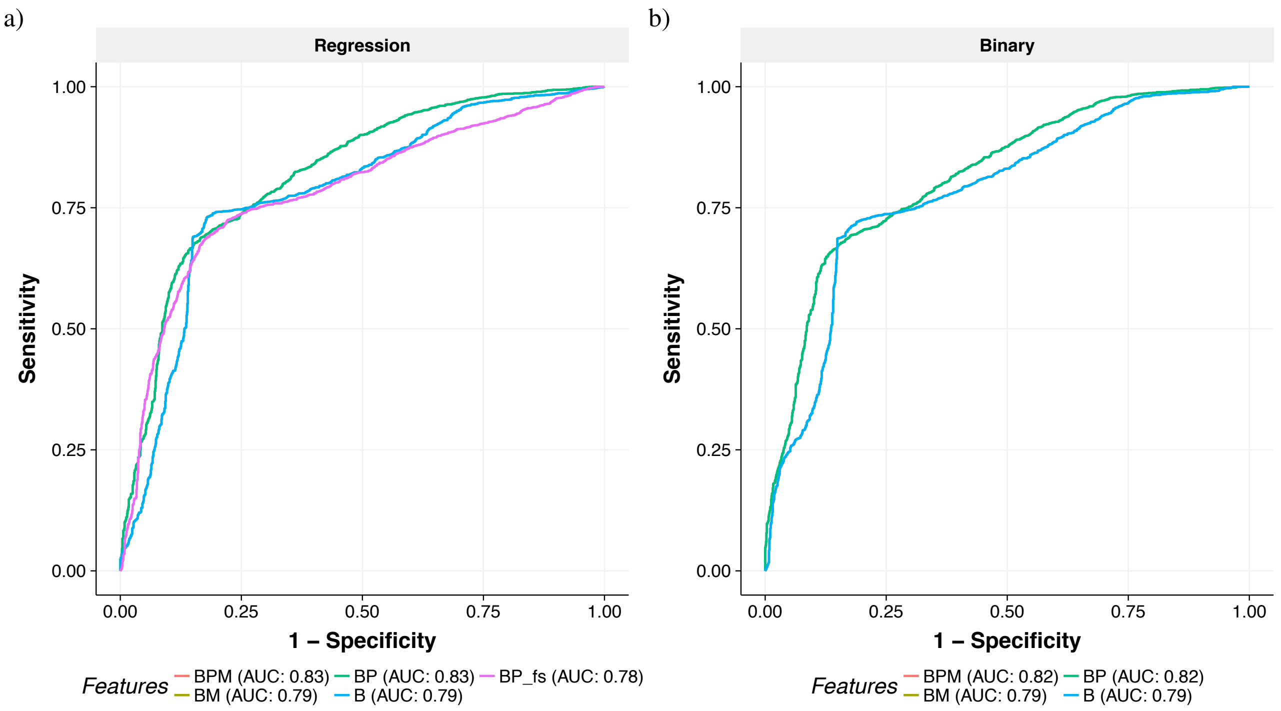

Figure 2. ROC curves for a) regression, and b) binary tasks comparing different sets of features to determine predictive value built on 162 plasma analytes with less than 1% missingness, rather than 15% in the main analysis, and 344 samples with no missing analyte measures. Hence this analysis shows the impact of imputation. As the results are very similar to the main analysis, albeit with metabolites have less impact as many were removed, and hence indicates the imputation used has minimal impact on our analysis.

Table 5: Means and standard deviation for protein and metabolite levels used in this study for either all samples (All) with or without CSF, only those samples that were CSF amyloid negative (AB-) or positive (AB+). The final two columns list the coefficient (B) and p-value (P) of a logistic regression of the analyte and amyloid status.

	**All**		**AB-**		**AB+**		**B**	**P**
	**mean**	**sd**	**mean**	**sd**	**mean**	**sd**		
A1MICRO	1.0854673	0.11615635	1.0679021	0.11009389	1.09657368	0.11710249	2.2	0.028
A2MACRO	0.05914279	0.09273091	0.04356849	0.10322482	0.06463167	0.09469178	2.4	0.056
AACT	2.49274118	0.15549316	2.48660546	0.16007136	2.49260799	0.16201143	0.23	0.74
AAT	0.43492947	0.093008	0.42975008	0.0879979	0.44074172	0.09424327	1.3	0.29
ACE	1.99140099	0.15875058	2.00428034	0.1695387	1.99475795	0.15717367	-0.37	0.6
ADIPNCTN	0.76161509	0.25308602	0.69503538	0.26854168	0.78485858	0.24481596	1.4	0.002
AFP	0.4341794	0.28153344	0.45573664	0.26591975	0.44065992	0.26822014	-0.21	0.61
AGRP	2.17809242	0.28016189	2.16126899	0.27535496	2.18832364	0.29734497	0.33	0.41
ANG2	0.65123698	0.1460979	0.64114059	0.14630985	0.65090264	0.15037432	0.45	0.56
ANGTNSNG	1.36005788	0.80027274	1.24011219	0.81738205	1.33421067	0.75547829	0.16	0.28
APOAI	-0.2260069	0.15854216	-0.2440324	0.16820733	-0.2205609	0.15744843	0.91	0.19
APOAII	2.68892516	0.11704129	2.71388689	0.13192162	2.68165603	0.10849066	-2.4	0.016
APOAIV	1.26802122	0.17332179	1.23793294	0.15221717	1.27360527	0.18810684	1.2	0.075
APOB	3.19199154	0.10371712	3.1847695	0.09678875	3.20576916	0.10835424	1.9	0.075
APOCI	2.55985673	0.12500084	2.54305605	0.1333353	2.57912222	0.12104766	2.3	0.012
APOCIII	2.13782823	0.15208605	2.13586025	0.14986792	2.14608921	0.1559752	0.44	0.55
APOD	2.19754797	0.12726707	2.20060622	0.12025343	2.20964799	0.11654912	0.66	0.49
APOE	1.70617827	0.19226899	1.78056958	0.16788475	1.68693259	0.20165905	-2.7	3.90E-05
APOH	364.003534	86.1011341	363.134454	84.2583723	374.384937	86.7120364	0.0016	0.24
AXL	1.21023769	0.1220698	1.20709975	0.10910058	1.20818686	0.13163203	0.07	0.94
B2M	0.29553103	0.13759686	0.29037479	0.11471358	0.3058864	0.15189715	0.81	0.33

BDNF	0.26997389	0.39618407	0.29210571	0.37174324	0.24886745	0.38606971	-0.3	0.31
BLC	1.44138965	0.22685461	1.43343798	0.2945512	1.44209795	0.18334977	0.17	0.73
BMP6	0.86586444	0.31588612	0.90114008	0.27096574	0.86468678	0.35419504	-0.34	0.32
BNP	2.99518433	0.34441698	2.87457975	0.38115971	3.02581473	0.33679565	1.2	0.00022
BTC	2.51493581	0.1859328	2.49426387	0.1917981	2.52985352	0.17771468	1.1	0.086
C3	0.24626678	0.08214229	0.2477774	0.0841889	0.24775368	0.07958401	-0.0036	1
CA199	1.14551726	0.44718634	1.0933721	0.48037568	1.16687364	0.43822449	0.35	0.15
CALCITNN	0.98541587	0.21407428	0.95377286	0.22465591	0.99132753	0.2369306	0.71	0.15
CD40	-0.122059	0.1310853	-0.1173462	0.1187837	-0.1193683	0.13551965	-0.12	0.89
CD40L	-0.5973717	0.48851383	-0.5338562	0.46849934	-0.6093672	0.49193029	-0.32	0.17
CD5L	3.53712919	0.15791127	3.57487487	0.13401899	3.52388331	0.17117295	-2	0.0057
CEA	0.2906855	0.25445431	0.28023689	0.22772276	0.30657126	0.28923365	0.36	0.39
CGA	2.67494896	0.39517223	2.75463336	0.37548295	2.61211172	0.41276561	-1	0.0022
CKMB	-0.4405222	0.24893863	-0.4308319	0.27344727	-0.4407189	0.22831112	-0.17	0.72
CLU	2.49239887	0.07578512	2.49125269	0.07641686	2.49831247	0.08054146	1.1	0.43
CNTF	1.73877334	0.3185044	1.7460995	0.29205356	1.75474812	0.31302401	0.093	0.8
CMPLMNFH	3654.33216	1184.8652	3640.41177	1125.71469	3778.62343	1230.19679	9.70E-05	0.3
CORTISOL	2.1674409	0.13199482	2.1587779	0.13169704	2.17663213	0.12618585	1.1	0.21
CPEPTIDE	0.38531565	0.20663387	0.38146	0.24101862	0.36831511	0.19045312	-0.31	0.57
CRP	0.12740782	0.53073142	0.25410521	0.43211227	0.08119854	0.56531302	-0.61	0.0041
CYSTATNC	3.12790571	0.10882697	3.12834605	0.09139031	3.12782778	0.12107251	-0.041	0.97
EGF	1.55022588	0.56056226	1.64378496	0.49611929	1.52938967	0.59180806	-0.37	0.071
EGFR	4.46466431	0.87367932	4.42184874	0.86083913	4.53556485	0.88271854	0.15	0.25
EPTHLDNA	-0.1239116	0.44742583	-0.0683466	0.43777588	-0.1374088	0.43265076	-0.37	0.16
EOTAXIN1	1.86665754	0.24455266	1.87353235	0.21283243	1.8620636	0.23379591	-0.22	0.65
EOTAXIN3	2.58505936	0.27145814	2.47467891	0.34957537	2.61655	0.23145863	1.8	2.90E-05
ESELECTN	7.11678445	3.0902904	7.20588235	2.76167151	7.14728034	2.85412552	-0.0074	0.85
FABP	0.55343018	0.36280857	0.61215698	0.32722326	0.54552234	0.35897544	-0.56	0.09
FACTRVII	2.77231689	0.1888721	2.78736319	0.15242261	2.77876004	0.17873755	-0.3	0.65
FAS	1.05091307	0.1398628	1.05607412	0.1557487	1.05032657	0.13511438	-0.28	0.72

FASL	1.77463823	0.25335839	1.80143034	0.23770538	1.78084343	0.28621435	-0.28	0.5
FETUINA	3.02105818	0.09416486	3.02538866	0.08360127	3.02218531	0.096088	-0.38	0.76
FGF4	2.37558426	0.35561302	2.34274664	0.35468084	2.37152075	0.3826021	0.21	0.49
FIBRINGN	6.35083922	1.5003623	6.33109244	1.38599214	6.37253975	1.67949423	0.017	0.82
FRTN	2.0063577	0.3850099	1.99718647	0.40891952	2.01364741	0.38490485	0.11	0.71
FSH	1.13659468	0.49731076	1.07645571	0.49281497	1.13109808	0.52146882	0.21	0.34
GH	-0.0644596	0.55018581	-0.0940744	0.52158287	-0.0821465	0.55168102	0.041	0.84
GROALPH	2.67229072	0.31710374	2.69725311	0.29503104	2.67287301	0.31420798	-0.26	0.48
GSTALP	1.10781343	0.37484006	1.12612219	0.35406684	1.05806121	0.37658395	-0.51	0.1
HAPTGLBN	0.18082403	0.48257204	0.19484815	0.40487788	0.1659541	0.53831408	-0.12	0.6
HBEGFGF	1.85172876	0.34339381	1.7825205	0.38169558	1.87061707	0.32294168	0.72	0.025
HCC4	0.7204577	0.21790984	0.73233824	0.2263777	0.7282264	0.19738796	-0.096	0.86
HGF	0.59793664	0.11730456	0.59768849	0.13810154	0.59520205	0.09820268	-0.19	0.84
I3	2.27466306	0.4718757	2.33731143	0.48627439	2.25176983	0.45969724	-0.38	0.11
ICAM	2.01392908	0.14797386	2.01342353	0.15623351	2.02002134	0.1352823	0.33	0.68
IGA	0.61246876	0.2256333	0.60535807	0.24457067	0.60745326	0.20713824	0.043	0.93
IGE	1.82644212	0.54410983	1.85579882	0.5536415	1.81311105	0.55314701	-0.14	0.49
INSLGFBP	1.96675511	0.21228329	1.93635008	0.21631009	1.9630649	0.19510776	0.67	0.24
IGM	0.24557917	0.27044901	0.31250378	0.26865891	0.2248182	0.24124214	-1.4	0.0024
IL13	1.59232313	0.17858917	1.6107479	0.13393813	1.59296188	0.19082879	-0.63	0.36
IL16	2.54548781	0.1617582	2.57816647	0.14228092	2.54072184	0.1565738	-1.7	0.031
IL18	2.4086544	0.17609512	2.41590487	0.20416815	2.39870987	0.15958239	-0.56	0.38
IL3	-1.6479139	0.28831026	-1.608359	0.24332829	-1.6583361	0.28507017	-0.69	0.1
IL6R	1.46453627	0.13495657	1.47684891	0.12347021	1.46545682	0.13091917	-0.7	0.43
IL8	1.01665959	0.19074979	1.04599504	0.17926761	1.00013657	0.19426262	-1.3	0.033
INSULIN	0.32227332	0.31155211	0.36972151	0.32011251	0.26870741	0.31698817	-1	0.0056
IP	2.64227401	0.18373151	2.66511924	0.15907774	2.62735439	0.18149356	-1.2	0.056
KIM1	-0.8212804	0.27307248	-0.8141526	0.24651175	-0.8322068	0.29281231	-0.23	0.56
LEPTIN	0.94396694	0.41769585	1.01953403	0.42917926	0.9073669	0.43216403	-0.62	0.022
LH	0.66859276	0.42449111	0.64859504	0.38663169	0.64436895	0.45624245	-0.023	0.93

A	2.30950624	0.60026998	2.26174395	0.61573852	2.32226092	0.57861074	0.17	0.36
MCP1	2.17619726	0.20820803	2.18062588	0.17062195	2.18196142	0.21469732	0.033	0.95
MCP2	1.53655557	0.16364706	1.55325025	0.17203374	1.52492289	0.16109797	-1	0.13
MCP3	0.89664988	0.38067771	0.90884882	0.33868916	0.90228084	0.40928553	-0.044	0.88
MCP4	2.57591099	0.12107684	2.57312605	0.13161965	2.56986716	0.12000496	-0.21	0.81
MCLNSF1	-1.4457142	0.13227635	-1.4462632	0.13792952	-1.4521449	0.13455846	-0.32	0.7
MDC	2.58414677	0.14696987	2.59538277	0.13903919	2.57933904	0.13253926	-0.88	0.29
MCRPHMIF	-0.3873697	0.29603809	-0.391244	0.27281986	-0.3733221	0.30809861	0.21	0.59
MI	3.53723795	0.24511225	3.56519328	0.25461621	3.52365264	0.24948823	-0.65	0.14
MCRPHIP1	2.2234064	0.09928672	2.21078756	0.10278085	2.22450527	0.09973606	1.4	0.23
MIPRT1B	2.16184982	0.20710953	2.1715805	0.20039626	2.14950285	0.21872342	-0.48	0.36
MCRPHIP3	1.80027194	0.21335781	1.81229395	0.20821785	1.79478531	0.21501667	-0.38	0.46
MMP1	0.09172051	0.25134711	0.04669798	0.23121541	0.11178962	0.25890147	1.2	0.022
MMP10	-1.3367585	0.20904752	-1.326114	0.18063952	-1.3241633	0.2445165	0.039	0.94
MMP2	3.58574438	0.24732054	3.59324445	0.12342125	3.55885406	0.35231073	-0.51	0.32
MMP7	0.18408799	0.24203915	0.19473992	0.20551491	0.19317611	0.27637984	-0.024	0.96
MMP9	2.14206254	0.25278481	2.14006286	0.19898527	2.12850335	0.27671856	-0.18	0.68
MM9T	2.3528764	0.20688657	2.34862756	0.20304324	2.35800849	0.19806795	0.24	0.67
MYLDPIF1	0.15434599	0.13288455	0.16161714	0.14429142	0.16039937	0.131068	-0.066	0.94
MPO	2.20489108	0.36547484	2.21219664	0.31823727	2.22930193	0.38216044	0.13	0.67
MYOGLOBN	1.3155294	0.22624737	1.3211474	0.21988819	1.3168805	0.22348811	-0.087	0.86
NTRPHGAL	2.42994025	0.15887031	2.4133879	0.13855437	2.44222586	0.16695268	1.2	0.11
NRCAM	0.35618424	0.20382109	0.37234235	0.15405344	0.33815067	0.26095912	-0.75	0.19
OSTEPNTN	1.33300184	0.21462317	1.35976034	0.20929159	1.33312511	0.21949228	-0.57	0.27
PAI1	1.66665622	0.27131684	1.70996504	0.27881778	1.65331088	0.27191744	-0.76	0.068
PAP	-0.7044179	0.21458452	-0.6771694	0.2403946	-0.7011956	0.21093951	-0.49	0.33
P	-1.7361399	0.30553089	-1.7603506	0.3086551	-1.7784523	0.32873174	-0.18	0.62
PLMNRARC	2.05979186	0.17004009	2.07595798	0.17343766	2.06433134	0.1636562	-0.42	0.53
PDGF	3.15145516	0.4883962	3.1735858	0.47005998	3.14422552	0.47606817	-0.13	0.58
PLGF	33.7379859	16.7360925	36.5323529	15.1310162	31.2085774	16.9561359	-0.02	0.0046

PPP	2.11366613	0.37169735	2.03258109	0.34857609	2.13625573	0.38896498	0.74	0.016
PRL	0.77763977	0.20596928	0.78897933	0.19760014	0.77305854	0.225166	-0.34	0.51
PRNSLNIN	0.55611527	0.33059189	0.55313361	0.396789	0.53589117	0.31380357	-0.15	0.65
PRNSLNTT	1.14666611	0.34319184	1.14450622	0.41523725	1.13016096	0.31409226	-0.12	0.71
PYY	2.00079993	0.30321458	1.92005647	0.31832355	2.04273661	0.3052522	1.3	0.00061
RCPTRFRD	0.59178133	0.26999299	0.56741193	0.24881879	0.61366335	0.26608197	0.68	0.12
RANTES	1.04270106	0.40103284	1.07713101	0.37197894	1.02709711	0.38853689	-0.34	0.24
RESISTIN	0.49094739	0.16101661	0.50772555	0.14179205	0.50457343	0.14679879	-0.15	0.85
SAP	1.32866405	0.1241719	1.32882126	0.11466747	1.33433916	0.11592264	0.42	0.67
SCF	2.42185917	0.18825799	2.43263891	0.18200118	2.4161641	0.19002416	-0.47	0.43
SRMGLTOT	3.515053	1.74470534	3.74386555	1.9212889	3.21882845	1.36920708	-0.21	0.0056
SHBG	1.75272403	0.20488696	1.73282479	0.20479085	1.75111632	0.21034638	0.42	0.43
SOD1	1.66031408	0.23552228	1.67188008	0.21188925	1.66571285	0.24591577	-0.11	0.81
SORTILIN	0.76172839	0.11657062	0.77192807	0.11241757	0.75681724	0.11615995	-1.1	0.24
TBG	1.77353678	0.09112822	1.7656258	0.08782845	1.77854506	0.09002093	1.6	0.2
TECK	2.26130553	0.20982407	2.27687891	0.22252595	2.26216649	0.22221673	-0.29	0.56
TSTSTRNT	0.13449039	0.4760308	0.15886706	0.51529813	0.12709159	0.47693282	-0.13	0.56
TFF3	-0.8108679	0.17208716	-0.8069347	0.15451936	-0.8026175	0.17011291	0.16	0.82
THP	-1.3766136	0.18331466	-1.349591	0.16901067	-1.3834543	0.18189172	-1.1	0.091
THRMBSP1	3.38567076	0.40193893	3.36935605	0.37055632	3.37518979	0.38782694	0.04	0.89
TSSINHM1	2.02936532	0.10508589	2.02815328	0.09955374	2.03134402	0.11348484	0.27	0.79
TM	0.88564161	0.11532035	0.87901311	0.1095622	0.8938013	0.11538033	1.2	0.25
TNC	2.74994154	0.19235818	2.70026345	0.18130835	2.76893456	0.18324085	2.1	0.0011
TNFALPHA	0.84265742	0.27696195	0.83164151	0.29643101	0.84723439	0.29083292	0.18	0.63
T	0.79199154	0.17224118	0.80143521	0.23314308	0.79507364	0.15981702	-0.18	0.76
THRMBPTN	2.1819788	0.98184834	2.30554622	0.92053752	2.07665272	1.00452573	-0.23	0.04
TNFRAILR	1.18516468	0.17107598	1.20863924	0.15843479	1.19248753	0.17381924	-0.57	0.39
TRNSFRRN	3.43511882	0.07745484	3.42404706	0.08324185	3.44116038	0.073261	2.9	0.049
TSH	0.2510773	0.31013408	0.29258966	0.25274097	0.2727767	0.28294886	-0.27	0.52
TTR	2.55177883	0.10722295	2.5786363	0.11090561	2.54870088	0.11117516	-2.4	0.018

VCAM	2.86448574	0.10634031	2.8596474	0.1052476	2.86709063	0.10938798	0.64	0.54
VEGF	2.79823959	0.12593158	2.78491109	0.11502341	2.80353586	0.13378867	1.2	0.2
VITRNCTN	2.9513555	0.06853542	2.96672765	0.0640102	2.94845799	0.07460511	-3.7	0.024
VKDPS	1.27872585	0.08992695	1.28055109	0.08286264	1.28709724	0.08931879	0.86	0.5
VWF	1.64312675	0.33811165	1.6468521	0.39397136	1.62797799	0.32743513	-0.16	0.63
AB40	151.558473	48.624629	156.179869	42.7897091	150.849932	49.9211208	-0.0024	0.32
AB42	36.5003795	11.0498447	38.9840238	11.8530481	35.0604352	10.3255157	-0.034	0.0018
HCAMPLAS	10.5431772	2.9197764	10.2433878	2.60743932	10.3782427	3.06162673	0.016	0.68
C0	33.553399	7.63479527	32.8991597	7.28254816	33.6728326	7.65195053	0.014	0.36
C10	0.37539014	0.3080933	0.35300974	0.28128988	0.39915543	0.38274047	0.47	0.26
C10.2	0.05840323	0.16224956	0.05147768	0.01695237	0.0514711	0.0178767	-0.021	1
C12	0.15574747	0.0615802	0.14783005	0.0585662	0.15931511	0.07271919	3	0.15
C14.1	0.07839637	0.02452118	0.07444538	0.02324217	0.08034165	0.02648701	10	0.043
C14.1.OH	0.01754672	0.003919	0.01765433	0.00389476	0.01754035	0.00397502	-7.3	0.8
C14.2	0.04524101	0.02064806	0.04347199	0.01811419	0.0460894	0.02244628	6.2	0.27
C16	0.1136924	0.02795479	0.11072269	0.02963619	0.11417321	0.02647273	4.7	0.27
C16.1	0.03395355	0.01363102	0.03216807	0.01518213	0.03501179	0.01444881	15	0.09
C18	0.05503801	0.01383902	0.0524317	0.0130897	0.0552139	0.01363437	16	0.068
C18.1	0.1487403	0.04225527	0.1442521	0.04479997	0.14980951	0.04081174	3.2	0.24
C18.2	0.06580798	0.02049201	0.06306723	0.01928806	0.06604633	0.01933854	8.3	0.17
C2	7.59435223	2.78900114	7.49016807	2.59950288	7.65111327	2.91704136	0.021	0.61
C3_metab	0.28266031	0.09085194	0.2952437	0.09253071	0.27645398	0.09042448	-2.2	0.069
C3.DC..C4.OH	0.07834504	0.03946263	0.08173027	0.05231584	0.07822167	0.03722667	-1.8	0.47
C4	0.23988913	0.21313892	0.24164706	0.09500193	0.22731819	0.08707083	-1.7	0.16
C6..C4.1.DC.	0.10416405	0.05217815	0.10367792	0.0541665	0.10634216	0.0600027	0.83	0.68
C5.DC..C6.OH	0.03629788	0.02428359	0.03422823	0.00919784	0.03624091	0.01068582	20	0.082
C7.DC	0.04878846	0.03363878	0.04694107	0.0187469	0.04872119	0.02047823	4.6	0.43
C9	0.03447607	0.06571751	0.03158246	0.01210559	0.03165526	0.01154492	0.53	0.96
lysoPC.a.C16.	153.096768	35.622614	150.605042	31.2139388	153.570254	34.5861536	0.0027	0.43
lysoPC.a.C16.	4.7886746	1.38291598	4.63361345	1.27902222	4.90340136	1.41965312	0.15	0.082

lysoPC.a.C17.	3.08960027	0.83265665	2.92529412	0.73341992	3.15428238	0.85707159	0.36	0.014
lysoPC.a.C18.	52.1535432	12.8988612	50.1563025	11.6353832	52.516216	12.8351516	0.016	0.094
lysoPC.a.C18.	32.9571883	8.33903969	31.6084034	7.80188569	33.6766529	8.67614969	0.031	0.03
lysoPC.a.C18.	38.3611238	12.2224325	37.4016807	11.6192635	38.657227	11.5101569	0.0096	0.33
lysoPC.a.C20.	2.94587625	0.95681864	2.88966387	0.83122121	2.93842093	0.92457239	0.062	0.63
lysoPC.a.C20.	9.79941834	3.14663723	9.60512605	2.80380095	9.92563281	3.29823909	0.033	0.36
lysoPC.a.C24.	0.26138535	0.07278157	0.26388235	0.07920261	0.25991712	0.06377334	-0.82	0.61
lysoPC.a.C26.	0.31669528	0.11641306	0.32873138	0.13200945	0.31581326	0.09961156	-1	0.3
lysoPC.a.C26.	0.28730175	0.13631004	0.29892437	0.16151561	0.28978905	0.12509376	-0.47	0.56
lysoPC.a.C28.	0.35750809	0.11171863	0.35786352	0.12935516	0.3592142	0.09386173	0.12	0.91
lysoPC.a.C28.	0.59152964	0.18377998	0.57553782	0.20186599	0.60285074	0.16995419	0.87	0.18
PC.aa.C24.0	0.13048546	0.0457773	0.13189159	0.04844651	0.13127436	0.04314827	-0.31	0.9
PC.aa.C28.1	2.8051202	0.73457967	2.64159664	0.70891536	2.85530983	0.71301129	0.44	0.0085
PC.aa.C30.0	3.38744228	1.27892631	3.26184874	1.22994623	3.39468087	1.3051521	0.084	0.36
PC.aa.C32.0	13.4546998	3.58756569	13.074958	3.74170696	13.5399604	3.58457059	0.037	0.26
PC.aa.C32.1	11.8271256	6.75364659	11.3584034	6.32998059	11.9660633	6.80035854	0.014	0.42
PC.aa.C32.3	0.66354029	0.19463618	0.63486555	0.18819819	0.68054388	0.1979325	1.3	0.039
PC.aa.C34.1	163.851447	30.8908303	163.302521	29.5177495	164.993949	30.1922394	0.0019	0.61
PC.aa.C34.2	162.129733	36.3886656	165.042017	40.2151829	162.493224	33.469749	-0.002	0.53
PC.aa.C34.3	16.6238476	5.45713169	16.0438656	5.09625737	17.1140882	5.61298208	0.038	0.082
PC.aa.C34.4	1.86180737	0.71620896	1.84493277	0.69033375	1.89676212	0.72048603	0.1	0.52
PC.aa.C36.0	2.74856113	1.54510711	2.92926891	1.56643994	2.61812098	1.43530583	-0.14	0.063
PC.aa.C36.1	67.294968	19.0214037	64.6655462	16.7160455	68.250702	19.6737988	0.011	0.09
PC.aa.C36.2	147.207364	30.9453039	149.495798	34.9192468	147.979068	29.598615	-0.0015	0.67
PC.aa.C36.3	129.544694	22.3388138	128.602521	22.4871167	131.956406	22.4592713	0.0068	0.18
PC.aa.C36.4	168.990725	30.9207894	167.462185	29.3542911	170.822643	31.9354024	0.0036	0.34
PC.aa.C36.5	22.6344862	14.9655128	22.7606723	12.9128783	23.1784937	16.7473446	0.0018	0.81
PC.aa.C36.6	0.83815561	0.40741076	0.84801681	0.40517299	0.84885522	0.43277207	0.0047	0.99
PC.aa.C38.0	3.60982261	1.10650964	3.6312605	1.17648396	3.52933896	1.10284058	-0.079	0.42
PC.aa.C38.3	53.7910022	15.0822103	53.7327731	15.0878563	53.6713941	13.6352945	-0.00031	0.97

PC.aa.C38.4	118.642642	25.3782605	116.966387	24.4595421	120.256897	26.3393591	0.005	0.25
PC.aa.C38.5	67.7717012	18.5129465	65.9672269	17.8942568	69.6499856	19.6729507	0.01	0.088
PC.aa.C38.6	80.8691285	28.5884827	81.6302521	26.7974941	80.508551	29.1653031	-0.0014	0.72
PC.aa.C40.2	0.45177824	0.11691283	0.45255462	0.11831978	0.45207466	0.11952635	-0.034	0.97
PC.aa.C40.3	0.76025095	0.16774048	0.74511765	0.17603381	0.76394282	0.1665915	0.67	0.32
PC.aa.C40.4	4.06225697	1.23668125	3.97092437	1.20961122	4.07229103	1.21869324	0.07	0.46
PC.aa.C40.5	11.8299322	3.36449964	11.5452941	3.39778213	11.9574332	3.33769968	0.037	0.27
PC.aa.C40.6	30.4707437	10.8736059	30.8378151	10.7459649	30.0201277	10.6892789	-0.0071	0.5
PC.aa.C42.0	0.65093482	0.20817819	0.63066387	0.20887935	0.65165328	0.21226121	0.48	0.38
PC.aa.C42.1	0.34079783	0.09914085	0.33203361	0.10085734	0.33868434	0.10025119	0.67	0.55
PC.aa.C42.2	0.29775286	0.08411796	0.28998319	0.07057788	0.2954693	0.08884273	0.82	0.56
PC.aa.C42.4	0.28366519	0.06368601	0.28238656	0.06388046	0.28333765	0.06707608	0.22	0.9
PC.aa.C42.5	0.3870606	0.10408333	0.38122689	0.10219725	0.3870389	0.10291252	0.56	0.61
PC.aa.C42.6	0.5573245	0.17480696	0.54795798	0.14117182	0.54978752	0.14020521	0.094	0.91
PC.ae.C30.0	0.3565078	0.0967874	0.34023529	0.09287624	0.35996708	0.09358409	2.4	0.062
PC.ae.C30.1	0.07729011	0.05461986	0.08215222	0.06218496	0.07772604	0.05140257	-1.4	0.47
PC.ae.C30.2	0.14261566	0.03318486	0.13834454	0.03625999	0.14490132	0.03145382	6.2	0.08
PC.ae.C32.1	2.30514923	0.64657572	2.24756303	0.6708014	2.30407257	0.62576846	0.14	0.43
PC.ae.C32.2	0.71655159	0.19545431	0.69089076	0.20047984	0.72419678	0.19819199	0.88	0.14
PC.ae.C34.0	1.62452902	0.46324035	1.55351261	0.44209589	1.63869653	0.48105053	0.41	0.11
PC.ae.C34.1	8.92676072	2.22196092	8.61672269	2.10882291	9.09011187	2.26734863	0.1	0.059
PC.ae.C34.2	10.4357484	2.9015973	10.0463025	2.71019727	10.6288761	3.02512367	0.07	0.078
PC.ae.C34.3	7.73619268	2.67123345	7.66647059	2.6962053	7.78061572	2.81934649	0.015	0.71
PC.ae.C36.0	1.03486094	0.28950528	1.00146427	0.24968336	1.03577167	0.30067004	0.44	0.28
PC.ae.C36.1	13.190473	3.07992221	12.5826891	2.58207304	13.4489447	3.31427035	0.096	0.014
PC.ae.C36.2	15.0006782	3.75399677	14.2478151	3.30548085	15.4582634	3.92801406	0.091	0.0047
PC.ae.C36.3	7.89951409	2.13059287	7.67806723	2.05046968	8.04197336	2.26489864	0.078	0.14
PC.ae.C36.4	16.4338309	4.24403066	16.3238656	4.54706501	16.4336263	4.34402093	0.0057	0.82
PC.ae.C36.5	12.4227879	3.753809	12.7566387	4.01146752	12.1739943	3.80035234	-0.038	0.18
PC.ae.C38.0	2.15695639	0.80454439	2.1522437	0.83340973	2.19863494	0.86984323	0.064	0.63

PC.ae.C38.2	1.61954387	0.44819135	1.56909244	0.39627482	1.63480913	0.48740523	0.32	0.2
PC.ae.C38.3	5.92788133	1.43165412	5.69798319	1.2434374	6.05035061	1.50239293	0.18	0.029
PC.ae.C38.4	14.2151492	3.3207542	13.8489916	3.41075751	14.3933028	3.60540184	0.045	0.17
PC.ae.C38.5	18.4035047	4.09298435	18.2853782	4.49093452	18.3683342	4.15761172	0.0046	0.86
PC.ae.C38.6	8.09026745	2.20894227	8.14	2.33638755	8.01169188	2.30327893	-0.024	0.62
PC.ae.C40.1	1.32378698	0.36104479	1.32165546	0.40742549	1.32659033	0.37208716	0.034	0.91
PC.ae.C40.2	1.97705039	0.51013289	1.91608403	0.50422832	2.0147408	0.52221106	0.38	0.091
PC.ae.C40.3	1.59890148	0.41765302	1.52806723	0.33639053	1.64467223	0.5029219	0.82	0.016
PC.ae.C40.4	2.57354273	0.56903594	2.48663866	0.5859908	2.60201534	0.62654954	0.32	0.096
PC.ae.C40.5	4.58066641	0.98252967	4.42705882	1.00475931	4.63798546	1.03563257	0.21	0.069
PC.ae.C40.6	5.07952826	1.34770366	4.99478992	1.36332771	5.06088628	1.35678645	0.036	0.66
PC.ae.C42.1	0.43206323	0.0940263	0.42457143	0.10663096	0.43552837	0.09618614	1.1	0.33
PC.ae.C42.2	0.55294772	0.13970801	0.53932773	0.14960927	0.55813819	0.13649641	0.97	0.24
PC.ae.C42.3	0.84087368	0.22264014	0.82959664	0.24877467	0.85383709	0.22780269	0.45	0.36
PC.ae.C42.4	0.89369394	0.23058705	0.86477311	0.25486715	0.90732946	0.23088714	0.77	0.11
PC.ae.C42.5	2.18154232	0.48241009	2.11673313	0.4863156	2.19379736	0.49325293	0.33	0.16
PC.ae.C44.3	0.13206568	0.03078428	0.12830252	0.03327634	0.13348126	0.03196938	5.2	0.16
PC.ae.C44.4	0.35427358	0.08931176	0.34181513	0.10034567	0.3625911	0.09029422	2.5	0.051
PC.ae.C44.5	1.52397818	0.44506008	1.46971429	0.48400911	1.55142174	0.45195199	0.4	0.12
PC.ae.C44.6	1.3087861	0.38369987	1.25689076	0.41346854	1.32254341	0.37897534	0.45	0.14
SM..OH..C14.	9.09363299	2.25047059	8.61932773	2.23307978	9.23688071	2.20217737	0.13	0.014
SM..OH..C16.	6.31319701	1.56435997	5.97285714	1.48778595	6.43893445	1.57403189	0.2	0.0083
SM..OH..C22.	49.834393	12.3210237	49.2361345	12.1627713	50.3820044	12.148898	0.0079	0.4
SM..OH..C22.	50.2698585	12.9896617	49.1588235	13.6807449	51.0076439	13.1845191	0.011	0.22
SM..OH..C24.	2.40507231	0.60179555	2.37932773	0.61445129	2.41740796	0.61920478	0.1	0.58
SM.C16.0	189.017838	41.2455371	182.764706	42.0997013	191.216651	40.4187616	0.0052	0.068
SM.C16.1	29.7568468	6.68747413	29.3840336	7.39615089	30.0893418	6.58208662	0.015	0.36
SM.C18.0	50.9722012	12.8136054	49.3067227	12.178668	51.5458897	12.6055073	0.015	0.11
SM.C18.1	25.3645049	6.94150244	24.7288235	7.16562536	25.7972526	6.80092385	0.023	0.17
SM.C20.2	1.32556683	0.39107516	1.25692437	0.37793539	1.36284185	0.39820779	0.73	0.018

SM.C24.0	48.9577672	12.3208878	49.8705882	12.5168927	48.8174255	11.7498028	-0.0073	0.43
SM.C24.1	179.658053	43.6077478	179.159664	45.8139168	180.383145	43.3014429	0.00063	0.8
SM.C26.0	0.39972257	0.09715473	0.39543698	0.10563221	0.39907097	0.09646613	0.37	0.74
SM.C26.1	0.67281226	0.18898265	0.65642017	0.19266021	0.67415811	0.18563311	0.52	0.4
Ala	499.300878	111.465966	501.983193	117.707479	498.442653	107.64993	-0.00029	0.78
Arg	169.21643	32.291973	170.983193	27.6193813	169.023542	33.5675976	-0.002	0.58
Asn	58.4073635	9.44541391	57.7344538	9.96370904	58.9554755	8.95838639	0.014	0.24
Asp	37.8726389	11.726265	37.4921008	11.6114526	37.3594996	12.0792554	-0.00094	0.92
Cit	46.3680222	11.8979144	45.5	11.6184614	46.3919222	11.9228769	0.0065	0.5
Gln	967.129827	152.1742	966.378151	157.364415	970.125274	146.214774	0.00017	0.82
Glu	101.688336	38.5213806	103.855462	36.6965908	97.6876208	36.141378	-0.0046	0.13
Gly	420.832481	108.938693	399.210084	92.0973945	416.8193	101.553093	0.0019	0.11
His	102.883167	15.5696153	103.561345	15.3306003	103.16104	15.7608818	-0.0016	0.82
Ile	85.066271	19.6353499	87.0571429	18.2553757	84.7759365	18.3652819	-0.0068	0.27
Lys	350.802337	77.0805125	348.537815	68.5680152	355.937764	79.6251037	0.0013	0.39
Met	31.2632033	6.31133566	31.287395	6.78757033	31.2360241	5.75110436	-0.0014	0.94
Orn	118.122211	32.0375989	114.333613	29.4056615	119.945626	31.0417183	0.0062	0.1
Phe	103.180415	21.4557089	103.221849	19.786381	102.235564	20.5522509	-0.0024	0.66
Pro	210.772135	66.9864476	207.94958	54.4998599	209.111368	67.2613676	0.00029	0.87
Ser	159.960872	31.0685459	159.890756	31.1172713	157.85449	32.6630471	-0.002	0.57
Thr	147.525151	32.261906	143.662185	29.1209605	149.485645	33.7099065	0.0058	0.11
Trp	71.8600566	15.3481473	72.7630252	15.9561787	71.9036802	14.7704266	-0.0037	0.61
Tyr	83.0034899	19.5831436	83.9142857	21.2591408	81.7902676	18.033015	-0.0057	0.32
Val	290.596797	58.4358955	294.97479	59.1726091	288.327853	55.5631895	-0.0021	0.3
ADMA	0.6599099	1.491138	0.5807563	0.13805648	0.60522911	0.14434506	1.2	0.13
Creatinine	101.03542	26.6719988	98.3420168	25.4289722	102.035442	26.9867337	0.0055	0.22
Kynurenine	3.19600244	1.02210387	3.24378151	0.95027203	3.19675321	1.06870402	-0.044	0.68
Putrescine	0.15804547	0.08742439	0.16297001	0.09055542	0.15716526	0.09476968	-0.65	0.58
Sarcosine	1.52241865	0.58090063	1.5587395	0.62749915	1.50841444	0.62655403	-0.13	0.47
Serotonin	0.53101548	0.35485138	0.61233731	0.35975456	0.50704181	0.37708002	-0.75	0.013

Spermidine	0.21084023	0.06600983	0.21016807	0.05560123	0.21262795	0.07021914	0.58	0.74
t4.OH.Pro	12.7012849	5.00318735	13.1782353	5.40678492	12.7482215	5.13139586	-0.016	0.46
Taurine	125.269519	32.7580932	125.991409	28.598161	125.037844	33.2287211	-0.00095	0.79
SDMA	0.66370072	0.16470602	0.64941177	0.15654801	0.67542119	0.16858915	1	0.16

Table 5: Means and standard deviation for protein and metabolite levels used in this study for either all samples (All) with or without CSF, only those samples that were CSF amyloid negative (AB-) or positive (AB+). The final two columns list the coefficient (B) and p-value (P) of a logistic regression of the analyte and amyloid status.

Supplementary References

1. H. D. Soares, W. Z. Potter, E. Pickering, M. Kuhn, F. W. Immermann, D. M. Shera, M. Ferm, R. A. Dean, A. J. Simon, F. Swenson, et al., Plasma biomarkers associated with the apolipoprotein e genotype and alzheimer disease, Arch. Neurol. 69 (10) (2012) 1310–1317.
2. L. St John-Williams, C. Blach, J. B. Toledo, D. M. Rotroff, S. Kim, K. Klavins, R. Baillie, X. Han, S. Mahmoudiandehkordi, J. Jack, et al., Targeted metabolomics and medication classification data from participants in the adni1 cohort, Scientific data 4 (2017) 170140.
3. L. M. Shaw, H. Vanderstichele, M. Knapik-Czajka, M. Figurski, E. Coart, K. Blennow, H. Soares, A. J. Simon, P. Lewczuk, R. A. Dean, E. Siemers, W. Potter, V. M.-Y. Lee, J. Q. Trojanowski, Alzheimer's Disease Neuroimaging Initiative, Qualification of the analytical and clinical performance of CSF biomarker analyses in ADNI., Acta Neuropathol. (Berl.) 121 (5) (2011) 597–609.
4. R. C. Petersen, P. Aisen, L. Beckett, M. Donohue, A. Gamst, D. Harvey, C. Jack, W. Jagust, L. Shaw, A. Toga, et al., Alzheimer's disease neuroimaging initiative (adni) clinical characterization, Neurology 74 (3) (2010) 201–209.
5. H. Ishwaran, U. B. Kogalur, E. H. Blackstone, M. S. Lauer, Random survival forests, The annals of applied statistics (2008) 841–860.
6. M. López-Ratón, M. X. Rodríguez-Álvarez, C. Cadarso-Suárez, F. Gude-Sampedro, et al., Optimalcutpoints: an r package for selecting optimal cutpoints in diagnostic tests, J Stat Softw 61 (8) (2014) 1–36.
7. R Core Team, R: A Language and Environment for Statistical Computing, R Foundation for Statistical Computing, Vienna, Austria (2016).
   URL https://www.R-project.org/
8. H. Ishwaran, U. Kogalur, Randomforestsrc: Random forests for survival, regression and classification (rf-src), R package version 1 (0).
9. H. Wickham, Ggplot2: Elegant Graphics For Data Analysis, Springer-Verlag New York, 2009.
10. X. Robin, N. Turck, A. Hainard, N. Tiberti, F. Lisacek, J.-C. Sanchez, M. Müller, proc: an open-source package for r and s+ to analyze and compare roc curves, BMC Bioinformatics 12 (2011) 77.
11. A. Kassambara, M. Kosinski, survminer: Drawing Survival Curves using 'ggplot2', r package version 0.3.1.999 (2017).
    URL http://www.sthda.com/english/rpkgs/survminer/
12. T. Benaglia, D. Chauveau, D. R. Hunter, D. Young, mixtools: An R package for analyzing finite mixture models, Journal of Statistical Software 32 (6) (2009) 1–29.
13. M. Hlavac, stargazer: Well-Formatted Regression and Summary Statistics Tables, Harvard University, Cambridge, USA, r package version 5.2 (2015).
    URL http://CRAN.R-project.org/package=stargazer
14. D. Attali, ggExtra: Add Marginal Histograms to 'ggplot2', and More 'ggplot2' Enhancements, r package version 0.6 (2016).
    URL https://CRAN.R-project.org/package=ggExtra
15. H. Wickham, stringr: Simple, Consistent Wrappers for Common String Operations, r package version 1.2.0 (2017).
    URL https://CRAN.R-project.org/package=stringr
16. H. Wickham, R. Francois, dplyr: A Grammar of Data Manipulation, r package version 0.5.0 (2016).
    URL https://CRAN.R-project.org/package=dplyr
17. H. Wickham, tidyr: Easily Tidy Data with 'spread()' and 'gather()' Functions, r package version 0.6.3 (2017).
    URL https://CRAN.R-project.org/package=tidyr
18. P. Solymos, Z. Zawadzki, pbapply: Adding Progress Bar to '*apply' Functions, r package version 1.3-1 (2016).
    URL https://CRAN.R-project.org/package=pbapply
19. G. Daróczi, R. Tsegelskyi, pander: An R Pandoc Writer, r package version 0.6.0 (2015).
    URL https://CRAN.R-project.org/package=pander
20. M. Dowle, A. Srinivasan, data.table: Extension of 'data.frame', r package version 1.10.4 (2017).
    URL https://CRAN.R-project.org/package=data.table
21. B. Auguie, gridExtra: Miscellaneous Functions for "Grid" Graphics, r package version 2.2.1 (2016).
    URL https://CRAN.R-project.org/package=gridExtra

22. M. K. C. from Jed Wing, S. Weston, A. Williams, C. Keefer, A. Engelhardt, T. Cooper, Z. Mayer, B. Kenkel, the R Core Team, M. Benesty, R. Lescarbeau, A. Ziem, L. Scrucca, Y. Tang, C. Candan, T. Hunt., Caret: Classification And Regression Training, r package version 6.0-73 (2016).
URL https://CRAN.R-project.org/package=caret
